# Supplementary material for: Heterocomplexes between the atypical chemokine MIF and the CXC-motif chemokine CXCL4L1 regulate inflammation and thrombus formation
Source: Cell Mol Life Sci. 2022 Sep 12;79(10):512. doi: 10.1007/s00018-022-04539-0 (PMC9468113; doi:10.1007/s00018-022-04539-0)
Supplement: Supplementary file 1 — Supplementary file1 (PDF 931 KB) [file 18_2022_4539_MOESM1_ESM.pdf]

Supplementary Files

Heterocomplexes between the Atypical Chemokine MIF and the CXC-Motif Chemokine CXCL4L1 Regulate Inflammation and Thrombus Formation

Markus Brandhofer[1,#], Adrian Hoffmann[1,2,#], Xavier Blanchet[3], Elena Siminkovitch[1], Anne-Katrin Rohlfing[4], Omar El Bounkari[1], Jeremy A. Nestele[4], Alexander Bild[4], Christos Kontos[5], Kathleen Hille[5], Vanessa Rohde[1], Adrian Fröhlich[1], Jona Golemi[6], Ozgun Gokce[6,7], Christine Krammer[1], Patrick Scheiermann[2], Nikolaos Tsilimparis[8], Nadja Sachs[9,10], Wolfgang E. Kempf[9], Lars Maegdefessel[9,10], Michael K. Otabil[1], Remco T.A. Megens[3,10,11], Hans Ippel[11], Rory R. Koenen[11], Junfu Luo[12], Bernd Engelmann[12], Kevin H. Mayo[11,13], Meinrad Gawaz[4], Aphrodite Kapurniotu[5], Christian Weber[3,7,11,10], Philipp von Hundelshausen[3,10,*], Jürgen Bernhagen[1,7,10*]

[1]Division of Vascular Biology, Institute for Stroke and Dementia Research (ISD), LMU University Hospital, Ludwig-Maximilians-Universität (LMU) München, 81377 Munich, Germany; [2]Department of Anesthesiology, LMU University Hospital, Ludwig-Maximilians-Universität (LMU) München, 81377 Munich, Germany; [3]Institute for Cardiovascular Prevention, LMU University Hospital, Ludwig-Maximilians-Universität (LMU) München, 80336 Munich, Germany; [4]Department of Cardiology and Angiology, University Hospital Tübingen, Eberhard Karls University Tübingen, Tübingen, Germany; [5]Division of Peptide Biochemistry, TUM School of Life Sciences, Technische Universität München (TUM); [6]Systems Neuroscience Group, Institute for Stroke and Dementia Research (ISD), LMU University Hospital, Ludwig-Maximilians-Universität (LMU) München, 81377 Munich, Germany; [7]Munich Cluster for Systems Neurology (SyNergy), 81377 Munich, Germany; [8]Department of Vascular Surgery, LMU University Hospital, Ludwig-Maximilians-Universität (LMU) München, 81377 Munich, Germany; [9]Department for Vascular and Endovascular Surgery, Klinikum rechts der Isar, Technische Universität München (TUM), 81675 Munich, Germany; [10]Munich Heart Alliance, 80802 Munich, Germany; [11]Cardiovascular Research Institute Maastricht (CARIM), Maastricht University, 6229 ER Maastricht, The Netherlands; [12]Vascular Biology and Disease Laboratory, Institute of Laboratory Medicine, Ludwig-Maximilians-Universität, LMU University Hospital, Ludwig-Maximilians-Universität (LMU) München, 81377 Munich, Germany; [13]Department of Biochemistry, Molecular Biology and Biophysics, Health Sciences Center, University of Minnesota, Minneapolis, MN, USA;.

Correspondence:

Jürgen Bernhagen, PhD
Professor and Chair of Vascular Biology
Institute for Stroke and Dementia Research (ISD)
LMU University Hospital (LMU Klinikum)
Ludwig-Maximilians-Universität (LMU) München
Feodor-Lynen-Straße 17, 81377 Munich, Germany
Tel.: 0049-89 4400 - 46151
E-Mail: juergen.bernhagen@med.uni-muenchen.de

Philipp von Hundelshausen, MD
Institute for Cardiovascular Prevention (IPEK)
LMU University Hospital (LMU Klinikum)
Ludwig-Maximilians-Universität (LMU) München
Pettenkofer Straße 8a/9, 80336 Munich, Germany
Tel.: 0049-89 4400 - 54359
E-Mail: Philipp.von_Hundelshausen@med.uni-muenchen.de

Contents

Supplementary Tables

Supplementary Figures

Supplementary Tables

Supplementary Table 1. Origin of thrombus tissue specimens and relevant clinical information on corresponding patients.

		Patient information					CV risk factors				Comorbidities				
#	Origin of thrombus	Age	Sex	Size [cm]	Weight [kg]	BMI	Hypertension	Diabetes	Smoking	Hyperlipidemia	CHD	Atrial fibrillation	PAD	COPD	Dialysis
1	AI/AFS/APF/AP	80	female	N/A	N/A	N/A	yes	no	no	yes	yes	yes	yes	no	no
2	AFC/AFS/APF/AP	73	female	N/A	N/A	N/A	yes	yes	no	yes	no	yes	yes	no	no
3	AB, AR, AU	64	female	N/A	N/A	N/A	no	no	ex	yes	yes	no	yes	no	no
4	AFC	72	male	175	50	16	no	no	ex	yes	no	no	yes	no	no

Table legend: Thrombus specimens were obtained via thrombectomy of the indicated blood vessels. The table provides an overview on relevant patient cardiovascular (CV) risk factors and comorbidities. BMI: Body mass index; CHD: coronary heart disease; PAD: peripheral artery disease; COPD: chronic obstructive pulmonary disease; AFS: *A. femoralis superficialis*, AFC: *A. femoralis communis*, APF: *A. profunda femoris*, AP: *A. poplitea*, AI: *A. iliaca*, AB: *A. brachialis*, AR: *A. radialis*, AU: *A. ulnaris*.

Supplementary Figures

Brandhofer*, Hoffmann* *et al.* Suppl Fig. 1

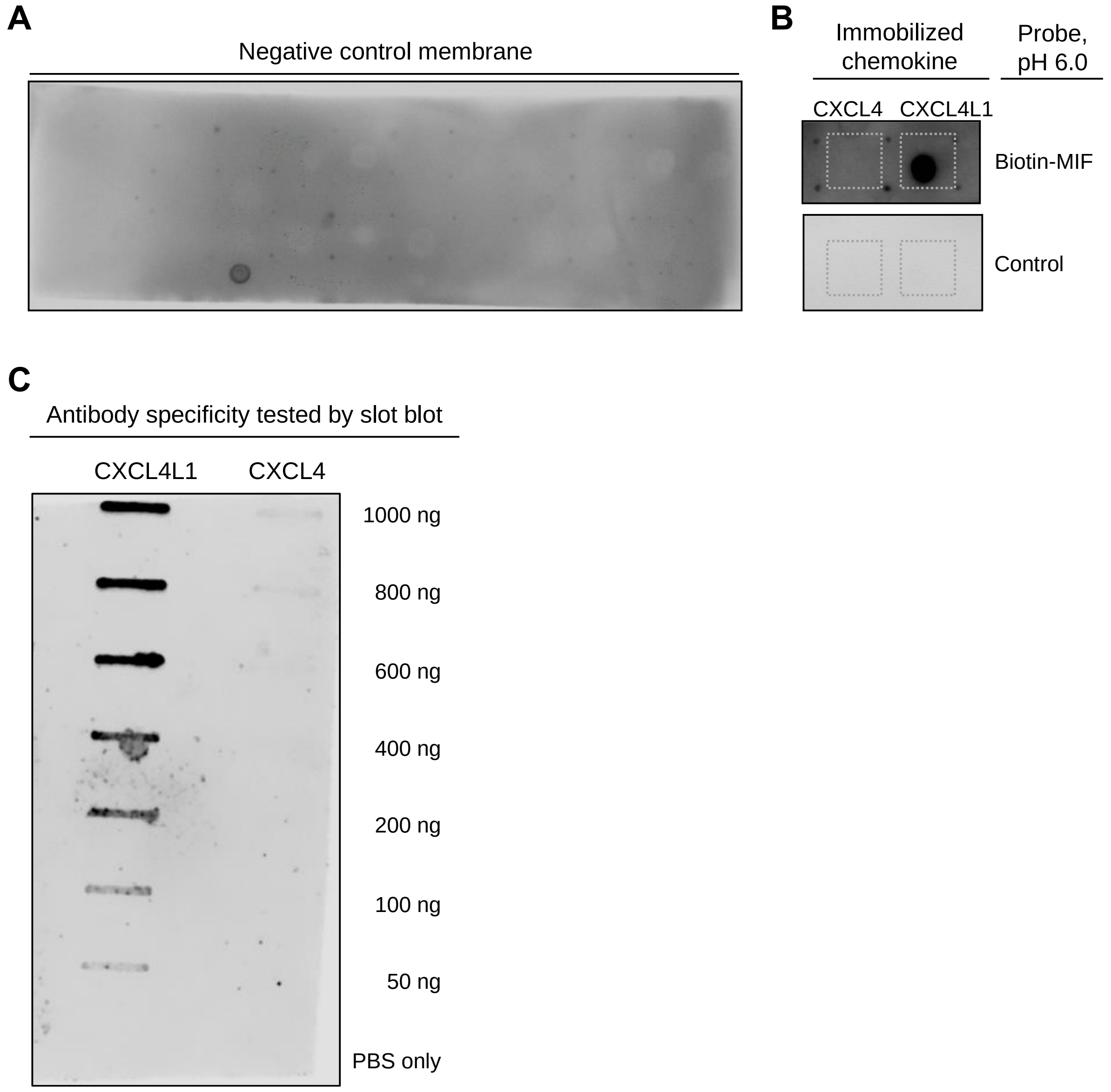

Supplementary Figure 1. Additional data for chemokine protein array. (**A**) Negative control membrane related to the experiment in **Figure 1**, incubated in buffer at pH 8.0 without biotin-MIF. (**B**) Close-up of membrane from a chemokine protein array experiment with a focus on CXCL4 and CXCL4L1. The membrane was incubated with biotin-MIF and the incubation was performed at pH 6.0. (**C**) Confirmation of specificity towards CXCL4L1 over CXCL4 of the anti-CXCL4L1 antibody used in this study. Varying amounts of recombinant CXCL4L1 or CXCL4 were applied on a nitrocellulose membrane via slot blot technique and developed against CXCL4L1.

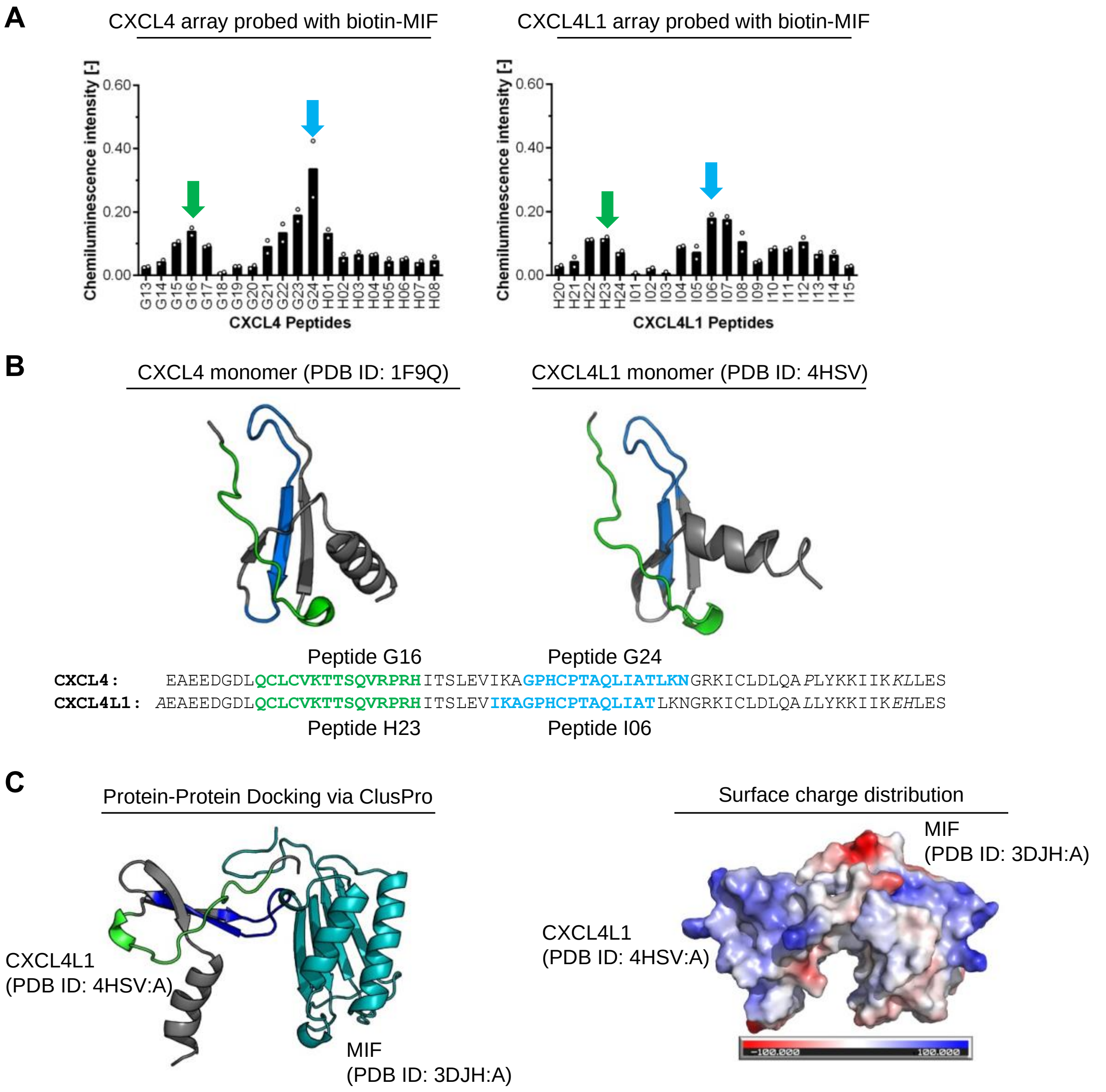

Supplementary Figure 2. Investigation of the MIF/CXCL4L1 interaction interface and *in silico* studies. (**A**) CelluSpot peptide array experiments, where overlapping peptides of CXCL4 (*left*) and CXCL4L1 (*right*) were spotted on an array and probed with biotin-MIF. Chemi-luminescence signal intensity indicates binding of biotin-MIF to the respective peptide. Arrows indicate peptides of interest that are most likely to be involved in the interaction with MIF. (**B**) Sequences of peptides identified in **A** are highlighted in the 3D structure of monomeric CXCL4 and CXCL4L1, showing their localization in the folded proteins. For both chemokines, these peptides of interest represent almost identical amino acid sequences, corresponding to highly similar regions of the protein. This indicates that not only the sequence but also the three-dimensional conformation of the chemokines might play a role in the interaction with MIF. Amino acid residues, in which CXCL4L1 differs from CXCL4 are in italics. PyMOL was used to visualize a CXCL4 (PDB ID: 1F9Q Chain A) and CXCL4L1 monomer (PDB ID: 4HSV Chain A). (**C**) To visualize the proposed MIF/CXCL4L1 complex, an unbiased *in silico* protein-protein docking approach was taken. The ClusPro 2.0 webserver was used to simulate a complex consisting of both a MIF and CXCL4L1 monomer. Depicted here is the highest-ranking docking result, with peptides identified in **A** to be potentially part of the interaction interface highlighted

in CXCL4L1. According to this *in silico* prediction, they are partially directed towards MIF, allowing parts of their sequences being involved in complex formation. PyMOL was used to calculate the surface charge distribution of these proteins (red: negatively charged; blue: positively charged), revealing an area of opposite charges in the proposed contact region of MIF and CXCL4L1 that partially matches the peptide array results.

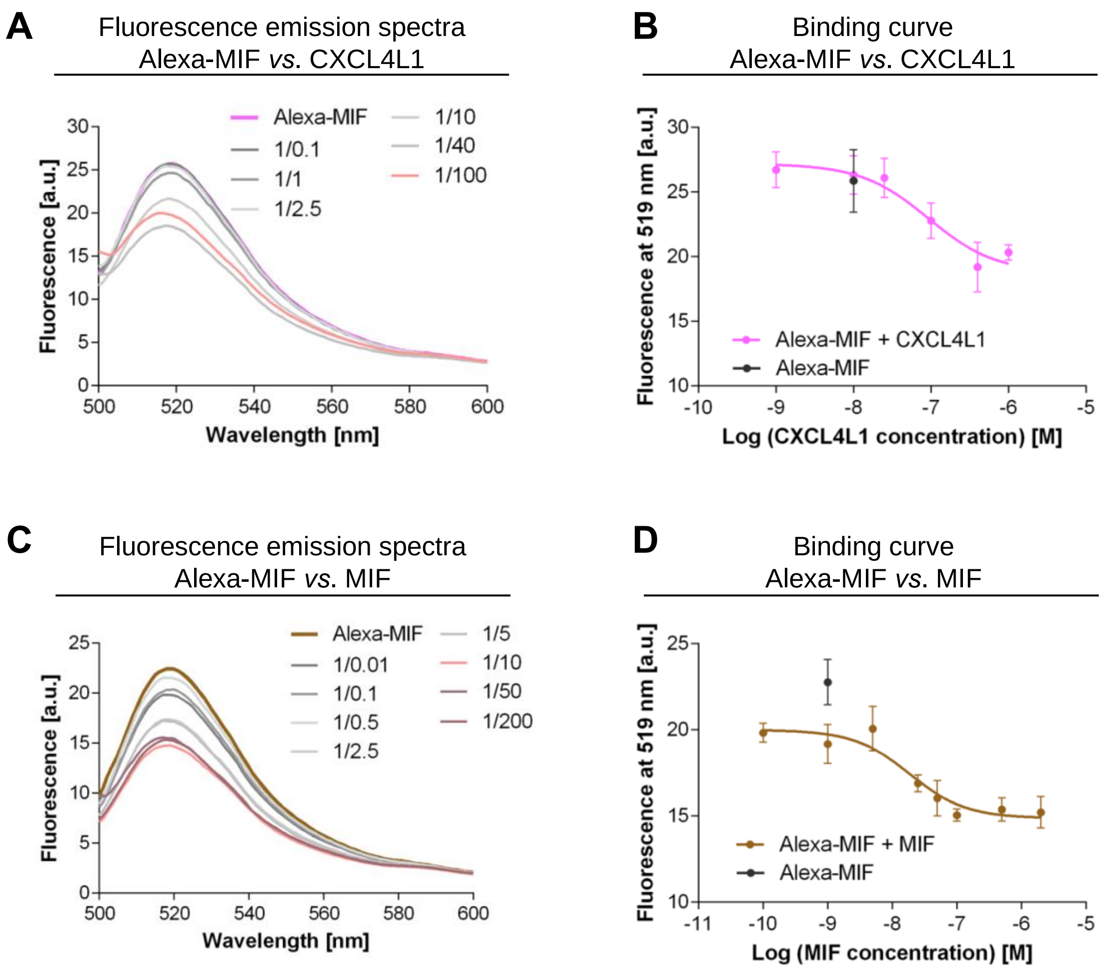

Supplementary Figure 3. Comparison of MIF/CXCL4L1 interaction to self-association of MIF as measured by fluorescence titration spectroscopy. (**A-B**) MIF binds to CXCL4L1. (**A**) Fluorescence emission spectra; fluorescently labeled MIF (Alexa-MIF, 10 nM) was titrated with increasing concentrations of unlabeled CXCL4L1 as indicated. (**B**) Binding curve; fluorescence emission at 519 nm was plotted against the concentration of CXCL4L1. Data shown are means ± SD from three independent titration experiments. The determined app. K_D is 86.4 ± 15.4 nM. (**C-D**) Homo-oligomerization of MIF. Same as (**A-B**) except that 10 nM Alexa-MIF was titrated with increasing concentrations of unlabeled MIF. (**C**) Fluorescence emission spectra. (**D**) Binding curve (app. K_D = 18.0 ± 5.1 nM). Data shown are means ± SD from three independent titration experiments.

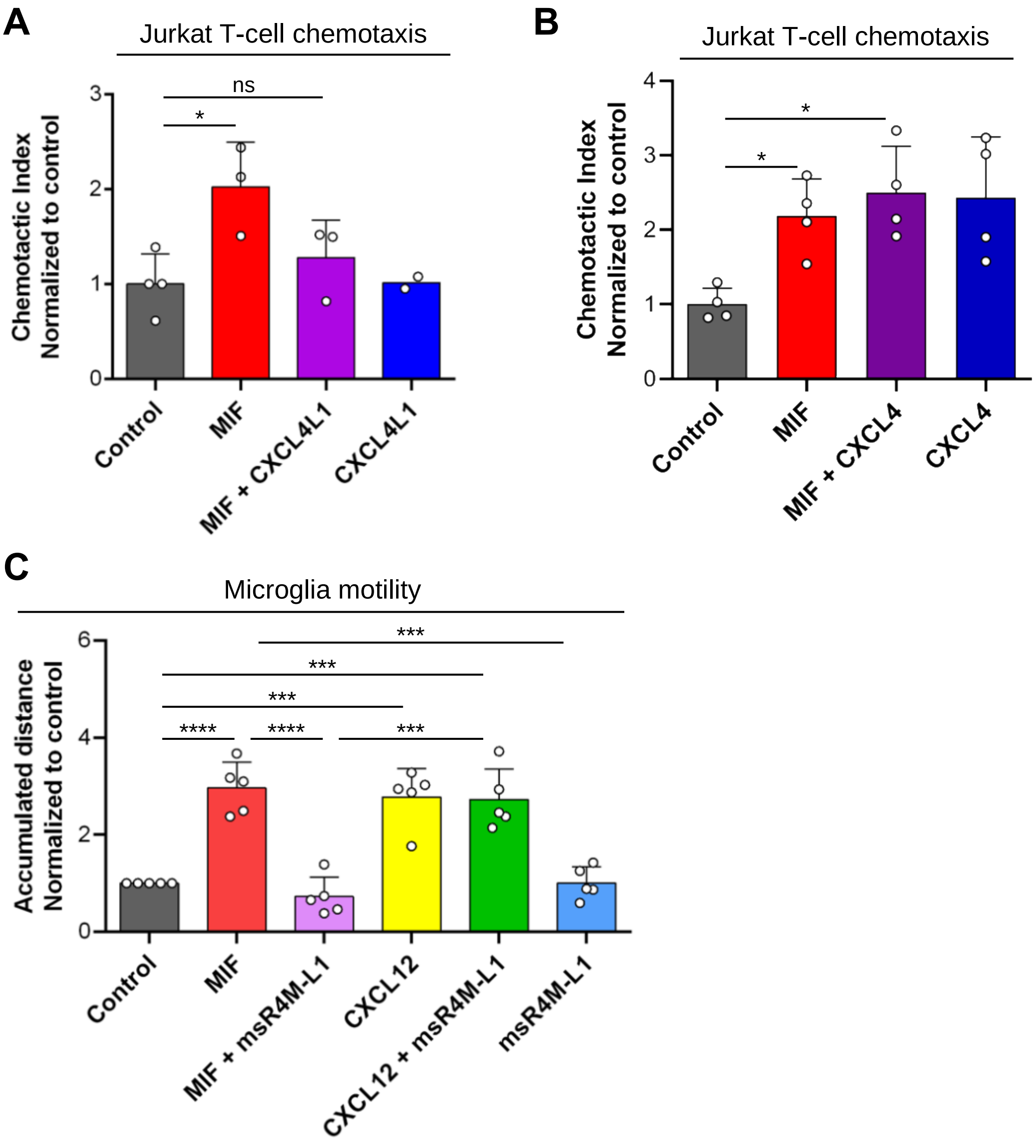

Supplementary Figure 4. Effects on cell migration in Jurkat T cells and microglia. (**A**) Effect of CXCL4L1 on MIF-mediated chemotaxis of Jurkat T cells as analyzed in a Transwell migration assay. Used concentrations: MIF: 16 nM, CXCL4L1: 32 nM; Data is presented as mean ± SD. n = 2-4 independent experiments. (**B**) Same as (**A**), except that co-incubation with CXCL4 was analyzed. Data is presented as mean ± SD. n = 4 independent experiments with duplicates each. (**C**) Quantification of murine microglia motility, based on the accumulated distance of GFP-positive microglia tracked during live cell imaging (n = 5). MIF was used at a concentration of 8 nM, the soluble CXCR4-mimicking peptide msR4M-L1 at 40 nM and the cognate ligand of CXCR4, CXCL12, at 16 nM. Data presented as mean ± SD. Statistical significance: *, $P < 0.05$; **, $P < 0.01$; ***, $P < 0.005$; ****, $P < 0.0001$.

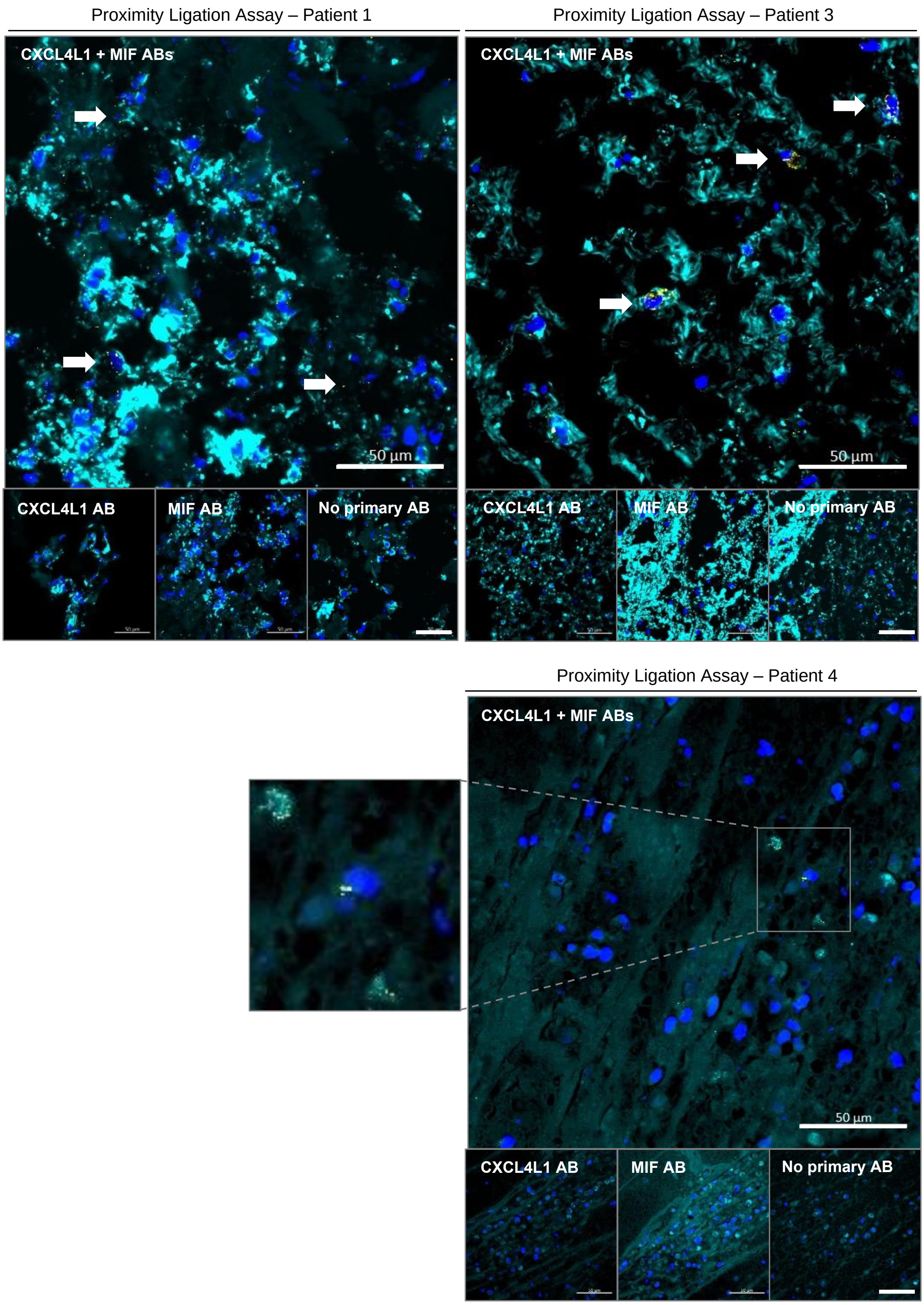

Supplementary Figure 5. MIF/CXCL4L1 heterocomplexes visualized by proximity ligation assay in three additional clinical thrombus tissue sections according to **Supplementary Table 1**. MIF/CXCL4L1 heterocomplexes appear as yellow punctate signals. For comparison, see

also **Figure 6**. Tissue was counterstained with fluorescent-labeled phalloidin (cyan); nuclei were stained by DAPI (blue). Stained tissue samples were imaged by CLSM; size bar: 50 µm. Negative controls are shown in bottom panels (CXCL4L1 antibody only, MIF antibody only, no primary antibody).

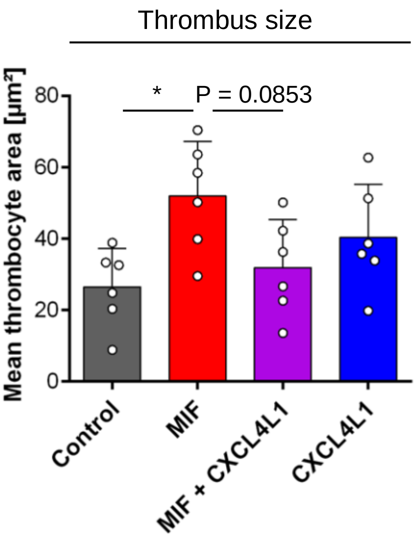

Supplementary Figure 6. Quantification of mean thrombus sizes from **Figure 5A**, showing a trend for CXCL4L1 inhibiting the MIF-mediated increase in thrombus size in samples, in which MIF and CXCL4L1 were pre-incubated together
